# Increased Serum Prolactin and Excessive Daytime Sleepiness: An Attempt of Proof-of-Concept Study

**DOI:** 10.3390/brainsci11121574

**Published:** 2021-11-28

**Authors:** Maria P. Mogavero, Filomena I. I. Cosentino, Bartolo Lanuzza, Mariangela Tripodi, Giuseppe Lanza, Debora Aricò, Lourdes M. DelRosso, Fabio Pizza, Giuseppe Plazzi, Raffaele Ferri

**Affiliations:** 1 Istituti Clinici Scientifici Maugeri, IRCCS, Scientific Institute of Pavia, 27100 Pavia, Italy; paola_mogavero@libero.it; 2 Department of Neurology I.C., Oasi Research Institute-IRCCS, 94018 Troina, Italy; fcosentino@oasi.en.it (F.I.I.C.); blanuzza@oasi.en.it (B.L.); mtripodi@oasi.en.it (M.T.); glanza@oasi.en.it (G.L.); darico@oasi.en.it (D.A.); 3 Department of Surgery and Medical-Surgery Specialties, University of Catania, 95123 Catania, Italy; 4 Division of Pulmonary and Sleep Medicine, Seattle Children’s Hospital, 4800 Sand Point Way, Seattle, WA 98105, USA; Lourdes.DelRosso@seattlechildrens.org; 5 IRCCS, Istituto delle Scienze Neurologiche di Bologna, 40139 Bologna, Italy; fabio.pizza@unibo.it (F.P.); giuseppe.plazzi@unibo.it (G.P.); 6 Department of Biomedical and Neuromotor Sciences, University of Bologna, 40126 Bologna, Italy; 7 Department of Biomedical, Metabolic and Neural Sciences, University of Modena and Reggio Emilia, 41125 Modena, Italy

**Keywords:** prolactin, hypersomnia, excessive daytime sleepiness, comorbidity, sleep disorders, observational study

## Abstract

The objectives of this study were: (1) to identify subjects with hyperprolactinemia in a clinical sample of patients; (2) to compare the neurologic, psychiatric, and sleep conditions found in patients subgrouped by excessive daytime sleepiness (EDS) and hyperprolactinemia; and (3) to identify patients with hyperprolactinemia and EDS not supported by the presence of any other neurologic, psychiatric, or sleep disorder, or substance/medication use. A retrospective chart review of inpatients was carried out in order to identify all patients in whom the prolactin (PRL) serum levels were determined. A total of 130 subjects were retrieved: 55 had increased levels of PRL, while the remaining 75 participants had normal PRL levels. EDS was reported by 32 (58.2%) participants with increased PRL and 34 (45.3%) with normal PRL. Obstructive sleep apnea or other sleep or neurologic/psychiatric conditions could explain EDS in all participants with normal PRL. Among subjects with increased PRL, eight had no other neurologic/psychiatric or sleep disorder (or drug) potentially causing EDS; these participants, at polysomnography, had time in bed, sleep period time, and total sleep time longer than those with EDS associated to another condition. These findings can be considered as a preliminary indication of a role of hyperprolactinemia in EDS and represent a basis for future controlled studies able to test this hypothesis in a reliable, objective, and methodologically more appropriate way.

## 1. Introduction

### 1.1. Experimental Rationale for a Possible Role of Prolactin in Sleep Regulation

Prolactin (PRL) is a peptide hormone mainly synthesized and secreted by the anterior pituitary gland; however, it can also be produced by the mammary gland, deciduous, prostate, skin, and possibly brain. Recently, the PRL receptor has also been detected in different regions of the brain, such as the cerebral cortex, the olfactory bulb, the hypothalamus, the hippocampus, and the amygdala [1]. New roles have been described for PRL, such as in neurogenesis, neurodevelopment and neuronal plasticity, sleep, learning, memory, and neuroprotection [2]. PRL is also involved in glial responses to cortical lesions caused by hypoxia; also, the number of oligodendrocytes of the corpus callosum seems to increase in relation to the levels of PRL [2].

Various monoaminergic neurotransmitters, hypocretins, corticotropin-releasing factor, and PRL are among the factors that seem to play an important role in stress-induced wakefulness and sleep changes [3,4]. Several studies have shown that PRL can induce REM sleep. It seems that stressful stimuli and conditions associated with large increases in PRL levels may be followed by an increased amount of REM sleep [3,5]. The underlying mechanism is still largely unclear, but there could be both a direct stimulatory effect of PRL on cholinergic neurons in the mesopontine tegmental area (involved in the induction of REM sleep), and an involvement of serotonergic circuits, since the raphe nucleus exerts an important control of REM sleep [3]. It has also been shown that sleep deprivation can induce a reduction in PRL levels (especially in the second part of the night), as well as in testosterone and cortisol levels [6,7]. 

These findings are important considering that higher concentrations of PRL appear to be associated with impaired cognitive performance and increased risk of depression [8]. In addition, preserving REM sleep is important for its multiple functions, such as brain maturation of newborns [9], memory consolidation [10], and regulation of emotions [11]. The concentration of PRL seems to be related to the sleep–wake cycle, as well as to the immune system [7]. Accordingly, hormones such as growth hormone, PRL, aldosterone, and catecholamines are the probable mediators of the migration of circulating T lymphocytes during sleep, which plays an important role in the modulation of immune reactions; in fact, during sleep, there seems to be a reduction in CD4+ and CD8+ lymphocytes [12].

### 1.2. Preliminary Clinical Reports Supporting a Possible Correlation between Hyperprolactinemia and Excessive Daytime Sleepiness

As seen above, the plasma concentration of PRL shows a sleep-dependent pattern, with higher levels during sleep and lower levels during the waking period [7]; however, there are not many studies in the literature on PRL or its eventual changes in the various sleep disorders in which excessive daytime sleepiness (EDS) can occur.

There are some data regarding obstructive sleep apnea (OSA), a disease that can occur with EDS and that is associated with hypoxemia and chronic sleep fragmentation, both of which could affect PRL secretion. A study on a series of 35 patients concluded that the prevalence of OSA in patients with prolactinoma is similar to that of obese subjects and not influenced by treatment; visceral obesity and higher BMI values, but not PRL levels, seemed to be the main factors involved in the onset of OSA in these patients [13]. Another study has instead shown that ventilatory treatment for OSA can normalize the release of PRL by restoring the pulse rate to values similar to those observed in normal subjects [14].

PRL is believed to protect the brain from hypoxia [15] and increased plasma PRL levels have been induced in animal models to protect cardiomyocytes by inhibiting cellular hypertrophy [16]. Thus, it is likely that the PRL variations observed in OSA are not related to the disease per se, but to the hypoxia often associated with this disorder.

There are only a few papers that explore the relationship of neuroendocrine hormones of the hypothalamus-pituitary-adrenal, hypothalamus-pituitary-thyroid, and hypo-thalamus-pituitary-gonadal axis with major depressive disorder also affected by OSA [17,18]. A recent report revealed an increase in cortisol and adrenocorticotropic hormone, an alteration of sleep structure with reduced slow-wave sleep, a reduction in PRL, and an increase in thyroxine during N1 and N2 sleep stages [18]. Moreover, a 42-year-old female has been reported with hypersomnia associated with menstrual periods, in whom hypersomnolence was found to be associated with marked elevations in the serum PRL level [19].

There seem to be no studies in the literature on hyperprolactinemia and hypersomnia or EDS; however, recent reviews have shown that sporadic cases of narcolepsy, a rare form of hypersomnia, have been reported in patients with sellar and pituitary adenomas, especially in pediatric age and regardless of anticancer treatment [20,21]. Indeed, cancer therapies can cause an alteration of the hypothalamus–pituitary-adrenal axis in pediatric age [22] and, consequently, hypersomnia.

### 1.3. Aims of the Study

Taking into consideration all the above data, it seems that despite a reasonably consistent rationale for a possible role of hyperprolactinemia in hypersomnia, the clinical data that would support this role are scarce and fragmentary. For this reason, the main scopes of this observational study were: (a) to identify subjects with hyperprolactinemia in a clinical sample of patients admitted to hospital for a relatively wide range of disorders, mainly neurologic, including sleep disorders; (b) to separate participants with a complaint of significant EDS from those without; (c) to compare the neurologic/psychiatric and sleep conditions found in participants with a complaint of EDS vs. those of participants without EDS, within each subgroup with or without hyperprolactinemia; (d) to identify patients with hyperprolactinemia (or not) and a complaint of EDS not supported by the presence of any other neurologic, psychiatric, or sleep disorder, or substance/medication use.

## 2. Materials and Methods

### 2.1. Subjects

A retrospective chart review of all patients admitted in the last 10 years at the Unit of Neurology I.C. of the Department for Brain Aging of the Oasi Research Institute–IRCCS, Troina, Italy, within which the Sleep Research Centre operates, was carried out. We then identified all patients in whom the PRL serum levels were determined (usually as a routine exam within the assessment of the pituitary function, whenever believed to be useful by the clinician). Patients of both sexes and with age 18 years or more were recruited regardless of the reason for their hospital admission. A total of 249 files were selected in this way, but we excluded from this sample all subjects with a significant cognitive impairment that would have interfered with their capacity to reliably report their subjective EDS, as reflected by a score of <22 at the Mini-Mental State Examination (MMSE) [23], which was obtained in all subjects. This information is routinely collected in the Department with a non-structured clinical interview and EDS is reported in the anamnesis if present and often, but not always, the Epworth sleepiness scale (ESS) [24] is collected and reported in the clinical file.

For each patient included in this analysis, the following data were routinely collected: sex, age, neurologic and psychiatric diagnosis, presence and type of sleep disorder, MMSE, serum PRL level, use of any drug acting at the central nervous system (CNS) level, complaint of subjective EDS and, whenever available, ESS score, hypocretin dosage in the cerebrospinal fluid (CSF), polysomnographically measured total sleep time, index of periodic leg movements during sleep (PLMS), apnea/hypopnea index (AHI), mean sleep latency and number of sleep-onset REM periods at the multiple sleep latency test (MSLT), brain imaging (magnetic resonance imaging in most cases, but with some computed tomography scans), and presence/absence of narcolepsy-like symptoms (i.e., sleep attacks, cataplexy, hypnagogic hallucinations, sleep paralysis, and automatic behaviors).

Polysomnography (PSG) and MSLT were recorded following standard practice rules [25], and standard criteria were also used for the scoring of sleep stages and apnea/hypopnea [25] or PLMS [26]. Fasting blood samples were taken in the morning from all patients, and the sera were used for estimations of routine investigation; subsequently, the serum PRL level was determined (chemiluminescence Liason, DiaSorin, Italy) in ng/mL.

All subjects included in this study had provided their informed consent for the use of their clinical and laboratory data for retrospective analyses, and the study was approved by the local Ethics Committee.

### 2.2. Statistical Analysis

Between group comparisons of continuous variables were carried out by means of the Student’s *t*-test and the nonparametric Mann–Whitney U test, as appropriate. Effect sizes were calculated with the formula r = z/√N; in agreement with Cohen [27], r values of 0.1, 0.3 and 0.5 indicate a small, medium, and large effect size, respectively. Comparisons of frequencies were performed by means of the Chi-square test or the Fisher exact *p*, as appropriate. Statistical significance was set at <0.05.

## 3. Results

A total of 130 subjects were retrieved who met the inclusion criteria set in the methods: 55 (14 males and 41 females, mean age 48.4 years, 16.58 SD) had increased levels of PRL (>21.4 ng/mL in males and >29.9 25 ng/mL in females, 56.7 ng/mL, 27.91 SD in females, mean PRL 51.7 ng/mL, 71.24 SD in males) and were labeled as high-PRL (hPRL) group, while the remaining 75 participants (28 males and 47 females, mean age 52.6 years, 13.61 SD, mean PRL 10.1 ng/mL, 6.79 SD in females, mean PRL 5.7 ng/mL, 4.62 SD in males) were labeled as normal PRL (nPRL) group. Sex composition (chi-square = 2.05, *p* = 0.152), age (Student’s *t* = 1.574, *p* = 0.118), and cognitive level (MMSE: hPRL mean 28.3, 2.60 SD vs. nPRL mean 28.6, 1.70 SD, Student’s *t* = 0.795, *p* = 0.428) were not significantly different between the two groups. Additionally, the number of subjects taking any drug was not significantly different between the two groups, with 35 out of 55 in the hPRL group and 52 out of 75 in the nPRL group (chi-square = 0.465, *p* = 0.495).

Among the narcolepsy-like symptoms collected from all subjects, we found a marginally statistically significant difference for the frequency of sleep attack, which was reported by 10 hPRL participants and five nPRL patients (chi-square 4.12, *p* = 0.042), while no statistically significant difference was found for the very few reports of cataplexy, hypnagogic/hypnopompic hallucinations, sleep paralysis, or automatic behaviors.

We subsequently subdivided these two groups into two subgroups each, based on their complaint of EDS, accompanied by significant daytime impairment in the personal, social, work, and cognitive or other areas of functioning, as reported in the clinical file. EDS was reported by 32 (58.2%, 10 males and 22 females) of hPRL participants, a percentage higher, but not significantly different from that found in nPRL subjects, 34 (45.3%, 17 males and 17 females) of whom reported a complaint of EDS (chi-square = 2.10, *p* = 0.147).

Table 1 details the sleep, neurologic, and psychiatric disorders found in the whole group of participants, subdivided by level of PRL and complaint of EDS. It is important to note that EDS was more frequently associated with sleep disordered breathing in nPRL patients than in hPRL subjects.

We also analyzed the frequency with which EDS was associated to a neurologic, psychiatric or sleep disorder able to explain it in both groups, hPRL and nPRL, and found this was possible in all participants in the nPRL group, but not in the hPRL group, among whom eight had no other disorder besides EDS (Fisher exact *p* = 0.0018).

Table 2 shows the clinical features and the laboratory findings retrieved with the retrospective chart analysis in the eight hPRL participants with a complaint of EDS and without another neurologic, psychiatric, or sleep disorder. Seven out of eight were females, their age ranged between 31 and 54 years, only two of them were taking drugs acting on the CNS, and CSF levels of hypocretin were available for two of them and were normal. Thyroid and the other pituitary hormones were found to be within the normal range in all subjects with the exception a slightly increased adrenocorticotropin in one subject (51.4 pg/mL, normal range 4.7–48.8). The ESS ranged from 15 to 22, none of the subjects showed a short total sleep time, nor a significant number of PLMS or OSA.

A MSLT was available for four participants who showed a short sleep latency in three cases and one sleep-onset REM period in two of them; in these two patients REM sleep latency was normal in the preceding night PSG. Brain imaging was normal in five patients, but showed signs of small vessel disease in one subject, mild cortical atrophy in another, and pituitary adenoma in the remaining participant. Six out of these eight subjects also reported narcoleptic-like symptoms, such as sleep attacks (five subjects), or hypnagogic hallucinations and automatic behaviors (one participant). One subject was under treatment with ranitidine and another with atorvastatine.

We subsequently assessed the presence of eventual differences between these eight hPRL patients with EDS, but without another neurologic, psychiatric, or sleep disorder and hPRL patients with a complaint of EDS associated with another sleep disorder, in terms of age, PRL levels, ESS, MMSE, and PSG parameters (available in seven and 18 participants, respectively); this comparison is reported in Table 3.

The main differences, which also were statistically significant, involved time in bed, sleep period time, and total sleep time, all definitely longer in the eight patients with EDS not associated to another sleep disorder and accompanied by medium-to-large effect sizes. Of note is that the percentage of REM sleep, PLMS index, and AHI (smaller in the eight patients) were also accompanied by a medium effect size, as well as PRL levels and minutes spent in REM sleep (tendentially higher in the eight patients), although without statistical significance (probably due to the small sample size). Finally, the eight hPRL patients with EDS, but without another neurologic, psychiatric, or sleep disorder were significantly younger than nPRL patients without sleep disorders (43.8 ± 7.57 vs. 54.0 ± 7.57 years; Mann–Whitney Z = −2.137, *p* = 0.033), while their MMSE was not statistically different (29.5 ± 0.76 vs. 27.8 ± 2.53; Mann–Whitney Z = 1.739, *p* = 0.082).

## 4. Discussion

With this observational, retrospective study, we found that, in a selected sample of patients admitted to hospital with neurologic/psychiatric and sleep disorders, increased levels of PRL is a relatively frequent finding when tested (55 out 130, 42.3%). Indeed, EDS tended to be more frequent among hPRL than nPRL patients, but the difference (58.2% vs. 45.3%, respectively) did not reach statistical significance. It is worth underlining here that almost all patients enrolled in this study had several neurologic, psychiatric, and sleep conditions and drug treatments able to induce EDS, thus blurring a possible effect of PRL levels. Perhaps a more controlled and powered analysis might be able to disclose such a difference with a more convincing statistical support.

In addition, we could demonstrate that EDS was associated with sleep-related breathing disorders in a number of subjects, which was higher in nPRL than in hPRL patients. Conversely, only in the hPRL a subgroup of patients in whom EDS was not associated to another neurologic/psychiatric condition or sleep complaint could be identified; this indirect evidence seems to further support the idea of a possible role of prolactin in EDS. As highlighted in the literature, some studies have suggested an association between OSA and PRL production, attributing this data to hypoxia [13,14,18]. However, based on what we have observed, it seems that PRL might really be associated to EDS, regardless of the concomitant presence of sleep-disordered breathing and hypoxemia.

The mechanisms by which PRL might be associated to EDS are poorly known and there is only evidence that PRL can promote REM sleep; indeed, large increases in PRL levels induced by stressful stimuli can cause an increase in the amount of REM sleep [3,5]. PRL, in fact, might directly stimulate cholinergic neurons in the mesopontine tegmental area (involved in the induction of REM sleep) and, possibly, also influence serotonergic pathways in the raphe nucleus that are also involved in the control of REM sleep [3]. It has also been shown that sleep deprivation can induce a reduction in PRL levels (especially in the second part of the night), as well as in testosterone and cortisol levels [6,7].

These findings provide a preliminary support to the original hypothesis of this study that increased levels of PRL might be associated with EDS and indicate the need of controlled studies directly assessing this hypothesis. In fact, it is clear that EDS has a multifactorial basis and that variables influencing it need to be controlled in order to disentangle the possible effect of PRL from that of a number of other factors influencing the level of sleepiness, in both physiology and pathology. EDS is highly prevalent and its causes are numerous, including inadequate sleep, sleep disordered breathing, circadian rhythm sleep–wake disorders, and central disorders of hypersomnolence (e.g., narcolepsy, idiopathic hypersomnia, and Kleine–Levin syndrome), but it also can represent a symptom of an underlying medical or psychiatric disorder [28]. We were not able to comprehensively control for all of these variables because of the retrospective nature of this study (this represents the main limitation of our analysis, among others), but we could demonstrate that EDS was associated with sleep disordered breathing more frequently in patients with normal levels of PRL than in those with increased levels. This may indicate that in the hPRL group the effect of sleep disordered breathing, one of the leading causes of EDS, might be less important and that other factors might be playing a key role, such as increased PRL. Again, this might constitute, even though indirectly, a support for our attempt of proof-of-concept and definitely represents the justification for more detailed and controlled studies in this field.

It is interesting to discuss why not all subjects with an increased level of PRL had a complaint of EDS in this study. For this reason, it is advisable to evaluate additional parameters with prospective studies, such as the duration of the disorder, the possible alteration in the secretion of other circadian hormones with possible impact on sleep, such as cortisol and thyroid hormones, and the sleep–wake pattern of these patients, with objective methods (such as actigraphy or PSG). Besides the obvious possible correlation with the magnitude of the increase of PRL (which was not evident in this study), it is important to emphasize that we took into consideration a subjective complaint of EDS and could not use a more objective measure, such as the MSLT, which was only available for very few subjects. However, the results of the comparison of the ESS scores were coherent with the subjective complaint of EDS showing significantly higher scores in patients reporting EDS. The evaluation of EDS by means of objective vigilance tests, in particular MSLT, would help identifying subjects with objectively assessed EDS who could be diagnosed as idiopathic hypersomnia and to establish if increased PRL may be a common finding in this diagnostic category or not. Moreover, other important factors could not be considered in this study, among which of particular importance is the psychological features of subjects who, however, were cognitively unimpaired and had all normal scores at the MMSE.

Even more importantly, in future studies, a more detailed evaluation of PRL levels needs to be performed, with repeated measures during the nictemeral cycle in order to assess its variability along the 24 h [29]. It should, however, be considered that in this study all PRL assessment were performed on blood drawn in the morning hours, typically between 8 and 10 a.m. We were unable to control for the menstrual cycle phase during which PRL was assessed in our premenopausal women (40 out of 88); however, PRL levels seem to show non-systematic changes during the course of the menstrual cycle, with peaks either during the ovulatory period or during the luteal phase [30]. During menopause PRL shows a progressive decline [31]. However, we used a cut-off value for the definition of increased PRL that is surely above the physiological peaks in premenopausal women. Furthermore, as mentioned before, sleep disorders can influence the levels of PRL which, in such a case, seem to be a secondary expression of a sleep disturbance and might not translate directly into an increased daytime sleepiness [32].

Finally, it is important to underline that we were also able to detect some differences in the sleep patterns of hPRL subjects with EDS and without another neurologic, psychiatric, or sleep disorder from those in whom EDS might be explained by the presence of these disorders. The small sample size of this analysis does not allow us to draw definite conclusions, but seems to indicate that in these patients there is a tendency to be sleepy during the day, notwithstanding a sufficiently long night sleep time. It will certainly be important, in the near future, to better characterize their sleep pattern from both the sleep architecture and microstructure points of view.

## 5. Conclusions

We were able to identify, among subjects with increased levels of PRL, a subgroup of patients in whom no other medical, neurologic, psychiatric, or sleep disorder (or drug) potentially causing EDS was present, but not among those with normal PRL. This cannot be considered a proof of causality between increased PRL and EDS, but provides an interesting support to our effort to obtain a proof-of-concept from these data and a basis for future controlled studies able to test in a reliable, objective, and methodologically more appropriate way the hypothesis of a role for hyperprolactinemia in EDS.

## Figures and Tables

**Table 1:** Sleep, neurologic, and psychiatric disorders found in the whole group of participants, subdivided by level of prolactin (PRL) and complaint of excessive daytime sleepiness (EDS).

	EDS (*n* = 66)			noEDS (*n* = 64)		
	hPRL (*n* = 32)	nPRL (*n* = 34)	^a^ Chi-Square or ^b^ Fisher Exact *p*	hPRL (*n* = 23)	nPRL (*n* = 41)	^a^ Chi-Square or ^b^ Fisher Exact *p*
	*n* (%)	*n* (%)		*n* (%)	*n* (%)	
*Sleep disorders*						
SRBD	13 (40.6)	27 (79.4)	10.4, 0.001 ^a^	2 (8.7)	8 (19.5)	0.220 ^b^
Insomnia	5 (15.6)	4 (11.8)	0.460 ^b^	2 (8.7)	5 (12.2)	0.508S ^b^
RLS/PLMS	4 (12.5)	5 (14.7)	0.540 ^b^	2 (8.7)	1 (2.4)	0.291 ^b^
Bruxism		1 (2.9)		1 (4.3)		
RBD				2 (8.7)		
CRSWD	2 (6.3)					
NT1	1 (3.1)					
NREM parasomnia		1 (2.9)				
Nocturnal seizures				1 (4.3)		
total	25	38		10	14	
*Neurologic Disorders*						
Migraine/Headache	2 (6.3)	2 (5.9)	0.670 ^b^	4 (17.4)	11 (26.8)	0.731, 0.771 ^a^
CVD	4 (12.5)	5 (14.7)	0.540 ^b^	5 (21.7)	6 (14.6)	0.347 ^b^
Seizures	1 (3.1)			3 (13.0)	2 (4.9)	0.242 ^b^
PD/LBD		2 (5.9)		2 (8.7)	1 (2.4)	0.291 ^b^
MCI		1 (2.9)			6 (14.6)	
TBI					1 (2.4)	
Cerebellar ataxia					1 (2.4)	
Optic neuritis					1 (2.4)	
						
total	7	10		14	29	
*Psychiatric Disorders*						
Anxiety/Depression	4 (12.5)	8 (23.5)	1.35, 0.246 ^a^	8 (34.8)	14 (34.1)	0.003, 0.959 ^a^
Personality disorder					1 (2.4)	
total	4	8		8	15	

SRBD = Sleep related breathing disorder; RLS = Restless legs syndrome; CRSWD = Circadian rhythm sleep-wake disorder; RBD = REM sleep behavior disorder; NT1 = Narcolepsy type 1; CVD = Cerebrovascular disease; PD = Parkinson disease; LBD = Lewy body dementia; MCI = Mild cognitive impairment; TBI = Traumatic brain injury.

**Table 2:** Clinical features and laboratory findings in high prolactin (hPRL) participants with a complaint of excessive daytime sleepiness and without another sleep disorder.

	Subject 1	Subject 2	Subject 3	Subject 4	Subject 5	Subject 6	Subject 7	Subject 8
Sex	F	F	F	F	F	F	M	F
Age, years	46	54	43	44	36	52	31	44
MMSE	30	29	28	30	29	30	30	30
Drug treatment	no	yes	no	no	yes	no	no	no
Prolactin, ng/ml	43.1	34.6	74.2	66.9	60.1	140.7	32.6	48.2
Pituitary hormones *	normal	normal	normal	↑ACTH	normal	normal	normal	normal
Thyroid hormones ^§^	normal	normal	normal	normal	normal	normal	normal	normal
Hypocretin, pg/ml					244.0			396.7
EDS	yes	yes	yes	yes	yes	yes	yes	yes
ESS	15	16	15	15	16	18	16	22
PSG, TST, min	457.5	391.5	495.5	424.0	593.0	422.0	523.5	405.0
PSG, PLMS index	0	1.8	3.5	0	0	0	0	0.6
PSG, AHI	0	5.8	6.3	1.2	5.6	1.3	0.4	0
MSLT, SL, min					4′06″	17′18″		0′12″
MSLT, SOREMPs					1	0		1
Brain imaging	normal	normal	SVD	cortical atrophy	pituitary adenoma	normal	normal	normal
Narcoleptic-like symptoms	sleep attacks			sleep attacks	sleep attacks, hypnagogic hallucinations, automatisms		sleep attacks	sleep attacks

* Human growth hormone (hGH), adrenocorticotropin (ACTH), thyroid stimulating hormone (TSH), luteinizing hormone (LH), follicle stimulating hormone (FSH); ^§^ triiodothyronine (T3) and thyroxine (T4); ↑ increased; MMSE = Mini-mental state evaluation; EDS = Excessive daytime sleepiness; ESS = Epworth sleepiness scale; TST = total sleep time; PLMS = periodic leg movements during sleep; AHI = apnea/hypopnea index; SL = sleep latency; PSG = polysomnography; MSLT = multiple sleep latency test; SOREMPs = sleep-onset REM periods; SVD = small vessel disease.

**Table 3:** Differences between the eight high prolactin (hPRL) patients with excessive daytime sleepiness (EDS), but without another sleep disorders and hPRL patients with a complaint of EDS associated to another sleep disorder.

	hPRL with EDS no Other Sleep Disorder (*n* = 8)		hPRL with EDS and Another Sleep Disorder (*n* = 24)		Mann–Whitney Test		
	Mean	S.D.	Mean	S.D.	Z	*p*-Value	Effect Size *r*
Age, years, whole group	43.8	7.57	44.3	17.17	–0.065	0.948	–0.01
Age, years, females	45.6	5.55	42.9	13.63	–0.098	0.922	–0.01
Age, years, males	31.0	-	46.8	22.63	-		
Prolactin, ng/mL, whole group	62.6	34.91	43.7	21.86	1.851	0.064	0.33
Prolactin, ng/mL, females	66.8	35.37	52.7	23.79	1.725	0.084	0.30
Prolactin, ng/mL, males	32.6	-	29.7	5.57	-	-	-
Epworth sleepiness scale	16.6	2.39	16.8	3.55	–0.116	0.908	–0.03
MMSE	29.5	0.76	29.4	1.01	0.000	1.000	0.00
Time in bed, min	540.4	66.70	458.1	92.48	2.270	0.023	0.45
Sleep period time, min	524.8	71.65	435.6	83.81	2.330	0.020	0.47
Total sleep time, min	464.7	82.33	371.9	77.96	2.209	0.027	0.44
Sleep latency, min	11.6	6.94	18.5	21.87	–0.393	0.694	–0.08
First REM latency, min	156.5	131.13	126.4	85.07	0.151	0.880	0.03
Stage shifts/hour	14.3	5.26	14.2	4.66	0.182	0.856	0.04
Awakening/hour	6.1	2.97	6.7	2.47	–0.212	0.832	–0.04
Sleep efficiency, %	85.7	8.20	81.7	9.22	0.999	0.318	0.20
WASO, %	11.7	8.11	14.4	8.59	–0.424	0.672	–0.08
Sleep stage N1, %	5.9	2.24	9.2	5.40	–0.999	0.318	–0.20
Sleep stage N 2, %	51.1	11.91	50.2	12.04	0.393	0.694	0.08
Sleep stage N3, %	12.0	8.74	11.3	7.27	0.030	0.976	0.01
Sleep stage R, %	19.3	6.59	14.9	6.50	1.362	0.173	0.27
NREM sleep, min	362.1	64.56	313.8	58.51	1.519	0.129	0.250
REM sleep, min	102.6	40.55	73.1	33.46	1.775	0.076	0.292
PLMS index	0.7	1.28	10.1	16.41	–1.407	0.100	–0.27
AHI	2.6	2.80	9.2	12.26	–1.806	0.150	–0.35

MMSE = Mini-mental state evaluation; AHI = Apnea/hypopnea index; PLMS = periodic leg movements during sleep; WASO = Wake Time After Sleep Onset.

## Data Availability

The data presented in this study are openly available in Mendeley at https://data.mendeley.com/datasets/2pxf4zdyhr/1 (accessed 18 October 2021).
